# Cross-cultural and clinical validation of the MDHAQ/RAPID3 questionnaire in electronic format for a Brazilian population of patients with rheumatoid arthritis

**DOI:** 10.1186/s42358-022-00278-9

**Published:** 2022-11-22

**Authors:** Ilka Benedet Lineburger, Claiton Viegas Brenol, Alice Silveira Goularte, Edila Penna Pinheiro, Vânia Naomi Hirakata

**Affiliations:** 1 Mestrado Profissional Em Pesquisa Clínica - Hospital de Clínicas de Porto Alegre, HCPA, Rua Ramiro Barcelos, 2350, Bairro Santa Cecília, Porto Alegre, RS CEP 90035-903 Brazil; 2 Universidade Federal do Rio Grande do Sul (UFRGS), Porto Alegre, Brazil; 3 Faculdade de Medicina, Universidade Federal Do Rio Grande Do Sul (UFRGS), Porto Alegre, Brazil; 4 Laboratório de Doenças Autoimunes (LABDAI), HCPA, Porto Alegre, Brazil

**Keywords:** Rheumatoid arthritis, RAPID3, Validation studies, Telehealth

## Abstract

**Background:**

Patients with rheumatologic diseases are monitored fundamentally through metric tools or index calculated from clinical data and patient exams, which allow us to assess the severity of the disease and guide the therapeutic decision. In rheumatoid arthritis (RA), for treatment to be optimized and considered effective, periodic assessment with composite disease activity index and a 'treat-to-target' approach is required. The Routine Assessment of Patient Index Data 3 (RAPID3) in the Multidimensional Health Assessment Questionnaire (MDHAQ) includes only three measures based on the central patient self-reported dataset and can be used in a 'treat-to-target' approach analogous to the Clinical Disease Activity Index (CDAI) and the Disease Activity Score 28-joints (DAS28). This tool, however, has not undergone cross-cultural or clinical validation in Brazil. In this research, we performed the MDHAQ cross-cultural and clinical validation for the Brazilian population of RA patients.

**Methods:**

The Portuguese version of the MDHAQ was created identically in an electronic questionnaire and underwent a cross-cultural validation process with 38 participants. Test–retest was performed in 29 patients. Further, a clinical validation with 129 Rheumatoid Arthritis patients was performed. Electronic MDHAQ was answered through an online platform. We also collected socioeconomic data as well as other clinical (CDAI, SDAI, DAS28) and functional (HAQ) scores during the face-to-face assessment of patients.

**Results:**

MDHAQ/RAPID3 maintained semantic, idiomatic, as well as conceptual and experience equivalence for the Brazilian population, with 92% acceptance of participants. It showed test–retest reliability, adequate internal consistency (Cronbach's α 0.85) and correlation of the scores obtained with adequate association with the DAS28 gold standard. RAPID3 also had high sensitivity (98%), adequate specificity (48%), high negative predictive value (92%) and negative post-test probability of 8%, attributes expected for a test tool for population screening.

**Conclusion:**

The use of MDHAQ/RAPID3 associated with traditional clinical measures can adequately allow for remote follow-up based on the 'treat-to-target' approach with performance comparable to the gold standard DAS28, being a viable tool in the sample of Brazilian patients with RA in the current context of telehealth.

## Introduction

In rheumatoid arthritis (RA), for an optimized and effective treatment to be achieved, periodic assessments with composite disease activity indices and a “treat-to-target” approach are required [1–5].

The Multidimensional Health Assessment Questionnaire (MDHAQ) is self-reported by the patient and developed in four components: (a) Routine Assessment of Patient Index Data 3—RAPID3, which includes 3 scores from 0 to 10 for functional activity, pain and global assessment of the patient, then composed on a scale from 0 to 30 and classified into categories of remission (≤ 3), low (3–6), moderate (6–12) and high (≥ 12) disease activity; (b) Fibromyalgia Assessment Screening Test 3—FAST3, which is a cumulative index based on Rheumatoid Arthritis Disease Activity Index—RADAI (score ≥ 16 = 1), symptom checklist (score ≥ 16 = 1), pain and/or fatigue (score ≥ 6 = 1), where a score ≥ 2 of 3 corresponds to more than 80% with the general symptoms scale based on the 2011 fibromyalgia diagnostic criteria; (c) PSYCH3, which includes sleep, anxiety and depression issues; (d) MEDI60, based on the symptom checklist, used for remote monitoring of adverse events and remote monitoring of patients [6, 7].

The RAPID3 can be used in a 'treat-to-target' approach in similarity to the Clinical Disease Activity Index (CDAI) and the Disease Activity Score 28-joints (DAS28) [8]. In 2020, the American College of Rheumatology (ACR) suggested strategies for the implementation of telehealth for patients with RA and among the clinical and functional assessment scores, RAPID3 did not needed adaptation for its use, since it is a self-reported questionnaire [9, 10].

In April 2022, the European Alliance of Associations for Rheumatology (EULAR) published points to be considered in telemedicine care for patients with rheumatological diseases, where it mentions that it can be used in the same pre-care assessment to help with referral to the specialty and prioritization of patients, for monitoring symptoms, disease activity and other consequences and assessment of the need for face-to-face consultation or therapeutic interventions [11].

The aim of this study was to proceed a cross-cultural and clinical validation of the MDHAQ/RAPID3 in electronic format for Brazilian Portuguese.

## Methods

The cross-cultural validation was performed following the guidelines proposed by Beaton [12]. As recommended by the mentioned author [12], for content validation, 38 participants (judges) were invited, including 13 RA patients, 8 relatives of patients with RA, 5 rheumatologists, 5 general practitioners and 7 nurses, all from the tertiary service of Hospital de Clínicas de Porto Alegre (HCPA). Participants were asked about the relevance and clarity of the tool. Adaptations and corrections were then submitted for the author’s appreciation.

The content validity index (CVI) was calculated to analyze the proportion of judges in agreement regarding relevance and clarity of the tool. A Likert-type scoring scale from 1 to 4 was used, with classification for the items as: 1 = Not relevant, 2 = Little relevant, 3 = Relevant, 4 = Very Relevant. The CVI score for the MDHAQ items was then calculated by the sum of agreement of items “3” and “4” by the participants, and then divided by the total number of responses [13, 14]. The questionnaire MDHAQ on its totality and RAPID3 were evaluated according to Polit and Beck [15], which consider the average values of the items calculated separately.

Participants were invited to answer a electronic questionnaire with reproduction of the MDHAQ items for consideration of conceptual equivalence, their general understanding and any cultural discrepancies or adaptations. In addition, to assess relevance and usability, we used the System Usability Scale (SUS) [16].

A further clinical validation was conducted in patients with RA according to the EULAR/ACR 2010 criteria [17] and access to digital media (text message or e-mail) consecutively invited from the Rheumatology Outpatient Clinics of HCPA during January to August 2021. Patients with inability to understand the instrument or incomplete information during data collection were excluded. All patients were previously instructed to complete the electronic questionnaire and assistance was available only when extremely necessary. *QuestionPro* platform has an obligatory response system that reinforce patient to complete missing data and not allowing to follow the next step and final submission if incomplete response, therefore missing data from the MDHAQ/RAPID3 online questionnaire in the study were not accounted.

Data was collected in part by the electronic questionnaire elaborated by *QuestionPro* platform, for later aggregation with the clinical database. Patients either received a weblink on their smartphones or responded to the electronic MDHAQ in a tablet provided by researchers during their rheumatologic appointment. Socioeconomic and demographic aspects and data regarding rheumatologic history (medication in use, time since diagnosis, laboratory tests) were also collected from the participants. Concomitantly to the week of their response to the electronic MDHAQ, patients completed an assessment of functional capacity estimated by the Health Assessment Questionnaire (HAQ) [18], calculation of the composite disease activity indexes (DAS28, CDAI and SDAI) performed by a rheumatologist and global assessment of disease determined by visual analogue scale (VAS) by both patient and physician.

Part of the patients answered the same electronic questionnaire at home through a weblink sent via e-mail or text message within 15 days from their first response. This sample was considered to perform a test–retest, as well for perception of possible difficulties encountered on the questionnaire.

A sample size was estimated considering a significance level of 5%, power of 80% and expected interclass correlation coefficient of 0.86, as referenced by Pincus et al. [19], with a total of 23 sample units for the test–retest. To assess the correlation of DAS28, SDAI and CDAI with MDHAQ/RAPID3 [20] considering a significance level of 1%, power of 90% and a correlation coefficient of 0.7, a sample size of 23 patients would be required. Estimating a Kappa of 0.65 and a frequency of 50% of correct answers for agreement between scores, the calculated sample size was 121 to ensure a power of 85%, using the validation study by Yokogawa for the Japanese population as a reference [21].

The validation and adaptation process of the MDHAQ (R877-NP2R) for a Brazilian population of patients with rheumatoid arthritis, has the authors authorization [22] and a version previously translated and back-translated for Brazilian Portuguese provided by the company RWS Life Sciences, responsible for managing the intellectual property of the author. The content of the electronic MDHAQ created by *QuestionPro* platform maintained the same format as its paper version. This study complies with the Declaration of Helsinki, was approved by the Ethics and Clinical Research Committee of the HCPA (Registration 2020-0602), and all steps were performed with patients Informed Consent.

### Statistical analysis

Analyzes were performed using the Statistical Package for Social Sciences program version 18.0 (SPSS Inc., Chicago–USA, 2009) and website http://vassarstats.net (Vassar College, New York–USA). Variables were evaluated by Shapiro–Wilk test and MDHAQ, HAQ, pain (VAS), physician and patient global assessments, number of tender and swollen joints, RAPID3, CDAI, SDAI did not presented normal distribution, therefore, their median and interquartile range (25–75%) were estimated and non-parametric tests were used. For DAS28, age and body mass index (BMI) mean and standard deviation were calculated. Categorical variables were described using absolute and relative frequencies. The internal consistency of the MDHAQ/RAPID3 components was assessed using Cronbach's alpha coefficient, we considered the values between 0.70 and 0.90 as accepted [23]. The reliability (test–retest) of the instrument was assessed using the intraclass correlation coefficient (ICC) that has the following cutoff: values less than 0.5 are indicative of poor reliability, values between 0.5 and 0.75 indicate moderate reliability, values between 0.75 and 0.9 indicate good reliability, and values greater than 0.90 indicate excellent reliability [24].

Spearman’s correlation coefficients were calculated for MDHAQ/RAPID3, clinical, laboratorial, and composite disease activity scores, as for physician and patient’s perceptions of their disease estimated by a visual analogue scale (0–10). The categories agreement for CDAI, SDAI, DAS28 were calculated using the linear and quadratic weighted Kappa and Kappa coefficients, with values between 0 and 0.2 considered as a poor agreement; between 0.21–0.4 reasonable; 0.41–0.6 moderate; 0.61–0.8 good and between 0.81 and 1.00 very good [25]. We also estimated the sensitivity, specificity and their respective 95% confidence intervals of RAPID3 to predict moderate/high activity and remission by the composite scores CDAI, SDAI and DAS28, as well as the negative post-test probability of RAPID3, using DAS28 as the gold standard. Categorical variables were compared using Fisher's exact test and continuous variables using Mann–Whitney and Kruskal Wallis. The significance level considered for all analyzes was 0.05.

## Results

### Content validation

For the first stage of the study, we counted with 38 participants with a mean age of 56 years (± 10), 13 years of education (± 5.6) and 30 (78%) were female.

The usability of the questionnaire, estimated by the System Usability Scale (SUS), was considered “good” for most patients (61%), familiars (50%) and “excellent” by nurses (42%), general practitioners (60%) and rheumatologists (80%). The mean was 70.6 (± 14.0) among all participants, with a minimum score of 32.5 and a maximum of 100. There was no association between SUS evaluation and the level education from participants (*p* = 0.092). Summing positive evaluation (good, excellent and best imaginable) the participants judgment was good, ranging from 60 to 100%. Regarding acceptance, 92% of the participants considered the MDHAQ a good instrument for following their rheumatologic symptoms.

According to Alexandre and Coluci [13] when assessing content validity index (CVI), the number of subjects must be also considered to define the cutoff point for agreement. In this study, a value not inferior to 0.78 was considered and this value was found on majority of items under consideration by professionals, with special emphasis on the evaluation of rheumatologist, greater public of interest in using the instrument, with a global CVI of 0.80 for MDHAQ and 0.97 for RAPID3.

### Test–retest validation

The intraclass correlation coefficient for the test–retest of the instrument MDHAQ/RAPID3 was performed for 23 participants and has the following results for the categories of MDHAQ ICC 0.96 (95%CI: 0.90–0.98) and RAPID3 ICC 0.89 (95%CI: 0.75–0.96).

### Clinical validation

The 129 patients included had mean age of 59 years (± 13), 108 were female (83.7%), median duration of disease were 13 years (25–75% interquartile range of 7–23), rheumatoid factor or Anti-CCP were present for 86.7% of the patients and 105 (81.4%) were using some synthetic disease-modifying antirheumatic drug (DMARD), 40 (31%) biological DMARD and 44 (34.1%) corticosteroids (Table 1).

**Table 1:** Characteristics of the validation study population (*n* = 129)

Characteristic	n (%)
Age in years	59 ± 13*
Female gender	108 (83.7%)
*Education level*	
Illiterate	1 (0.8%)
Up to 1st grade	82 (64.6%)
Up to 2nd grade	36 (28.3%)
Superior	8 (6.3%)
BMI	26 ± 4*
Disease duration (years)	13 (7–23)**
RF or anti-CCP positive	111 (86.7%)
Using corticoid	44 (34.1%)
Using conventional synthetic DMARD	105 (81.4%)
Using biologic agents	40 (31.0%)
Rheumatoid arthritis core data set measures	
*Patient measures*	
MDHAQ (0–3)	1.0 (0.4–1.5)**
HAQ (0–3)	1.1 (0.6–1.7)**
Pain VAS (0–10)	6.0 (3.5–8.0)**
Global Assessment (0–10)	5.0 (2.0–7.5)**
*Physician measures*	
Tender 28-joint count	2.0 (0.0–4.5)**
Swollen 28-joint count	1.0 (0.0–4.0)**
Evaluator global assessment of disease activity (0–10)	3.0 (0.0–5.0)**
*Laboratory measures*	
Erythrocyte sedimention rate (mm/h) [*n* = 128]	26.5 (15.0—44.0)**
C-reactive protein (mg/dl) [*n* = 127]	5.4 (2.2—13.0)**
*Composite disease activity index*	
RAPID3	15.0 (8.8–19.6)**
CDAI	11.3 (4.0–20.0)**
SDAI [*n* = 127]	12.1 (4.7–21.8)**
DAS28 (ESR)	3.8 ± 1.6*

*Mean and Standard Deviation; ** Median and Interquartile range

BMI: Body Mass Index; RF = Rheumatoid Factor; Anti-CCP = Anti Cyclic Citrullinated Peptide; DMARD = Disease Modifying Antirheumatic Drugs; MDHAQ = Multidimensional Health Assessment Questionnaire; HAQ = Health Assessment Questionnaire; VAS = Visual Analog Scale; ESR = Erythrocyte sedimention rate; RAPID3 = Routine Assessment of Patient Index Data 3; CDAI = Clinical Disease Activity Index; SDAI = Simple Disease Activity Index; DAS28 = Disease Activity Score 28

The internal consistency of the RAPID3 was estimated using Cronbach's α, and its general evaluation presented a coefficient of 0.85. The effect on Cronbach's α when individual component was assessed was 0.90 for the MDHAQ functional score, 0.72 for pain and 0.68 for the patient's global assessment. The internal consistency of the functional part of the MDHAQ had a Cronbach's α coefficient of 0.91, and there was no discrepant item or whose removal would cause a relevant impact on the internal consistency of the questionnaire.

The median time to complete the electronic MDHAQ was 20 min (25–75% interquartile range of 15–28) and for the RAPID3 5 min (25–75% interquartile range of 3–6).

The correlation of RAPID3 with CDAI and SDAI was 0.88 (*p* < 0.001) and 0.89 (*p* < 0.001), respectively, and 0.53 for DAS28 (ESR) (*p* < 0.001), other correlations are shown in Table 2.

**Table 2:** Inter-component correlation coefficients (Spearman's correlation)**

	RAPID3	CDAI	SDAI	DAS28	TJC28	SJC28	VAS	PGA	EGA	MDHAQ	CRP
CDAI [n = 128]	0.61										
SDAI [n = 127]	0.61	0.99									
DAS28 (ESR)	0.53	0.88	0.89								
TJC28	0.52	0.88	0.87	0.84							
SJC28	0.41	0.82	0.82	0.74	0.78						
VAS	0.90	0.58	0.58	0.49	0.45	0.40					
PGA	0.64	0.78	0.80	0.68	0.56	0.47	0.64				
EGA	0.52	0.87	0.86	0.76	0.73	0.69	0.44	0.71			
MDHAQ	0.78	0.44	0.43	0.40	0.44	0.31	0.55	0.38	0.41		
CRP [n = 127]	0.25	0.28	0.37	0.50	0.26	0.20*	0.24	0.32	0.21*	0.19*	
ESR [n = 128]	0.12 Δ	0.23	0.28	0.58	0.22*	0.19*	0.09 Δ	0.18*	0.22*	0.13 Δ	0.60

TJC28 = Tender 28-joint count; SJC28 = Swollen 28-joint count; PGA = Patient's global assessment; EGA = Evaluator's global assessment; MDHAQ = Multidimensional Health Assessment Questionnaire; VAS = Visual analog scale; ESR = Erythrocyte sedimention rate; CRP = C-reactive protein; RAPID3 = Routine Assessment of Patient Index Data 3; DAS28 = Disease Activity Score 28

*The correlation is significant at the level of 0.05 (two-tailed), in the others the correlation is significant at the level of 0.01 (two-tailed)

**The number of subjects analyzed is 129, except when described in its component

Δ Not significant, *p* > 0.05

The agreement analysis was performed according to the disease activity categories (Table 3), a grouped analysis for patients with remission/low activity and moderate/high activity in the different composite disease scores were also performed to better characterize the groups in patients with controlled disease and active patients (Table 4).

**Table 3:** Correlation between RAPID3 and the categories of composite disease activity indices (CDAI, SDAI e DAS28)

	RAPID3				
	Remission	Low	Moderate	High	Total
*CDAI*					
Remission	**8**	9	4	6	*27*
Low	4	**1**	9	16	*30*
Moderate	2	0	**9**	33	*44*
High	0	0	2	**26**	*28*
Total	*14*	*10*	*24*	*81*	*129*
Kappa 0.132 (95%CI = 0.04–0.21)					
Linear weighted kappa = 0.29 (95%CI = 0.19–0.39)					
Quadratic weighted kappa = 0.43 (95%CI = 0.31–0.54)					
*SDAI*					
Remission	**9**	9	4	5	*27*
Low	3	**1**	8	16	*28*
Moderate	2	8	**10**	38	*24*
High	0	16	2	**20**	*79*
Total	*14*	*10*	*24*	*79*	*127*
Kappa 0.118 (95%CI = 0.03–0.20)					
Linear weighted kappa = 0.28 (95%CI = 0.19–0.38)					
Quadratic weighted kappa = 0.43 (95%CI = 0.31–0.55)					
*DAS28 (ESR)*					
Remission	**8**	9	6	10	*33*
Low	4	**1**	3	4	*12*
Moderate	2	0	**12**	38	*52*
High	0	0	3	**28**	*31*
Total	*14*	*10*	*24*	*80*	*128*
Kappa 0.162 (95%CI = 0.06–0.25)					
Linear weighted kappa = 0.32 (95%CI = 0.22–0.42)					
Quadratic weighted kappa = 0.44 (95%CI = 0.32–0.56)					

RAPID3 = Routine Assessment of Patient Index Data 3; CDAI = Clinical Disease Activity Index; SDAI = Simple Disease Activity Index; DAS28 (ESR) = Disease Activity Score 28; Bold: number of coincidental categories among analyzed scores; Italic: total number in respective categories

**Table 4:** Agreement between the categories grouped in RAPID3, CDAI, SDAI and DAS28

	RAPID3		
	Remission/low	Moderate/high	Total
*CDAI*			
Remission/low	**22**	35	*57*
Moderate/high	2	**70**	*72*
Total	*24*	*105*	*129*
Kappa 0.38 (95%CI = 0.24–0.52)			
*SDAI*			
Remission/low	**22**	33	*55*
Moderate/high	2	**70**	*72*
Total	*24*	*103*	*127*
Kappa 0.39 (95%CI = 0.25–0.54)			
*DAS28 (ESR)*			
Remission/low	**22**	23	*45*
Moderate/high	2	**81**	*83*
Total	*24*	*104*	*128*
Kappa 0.52 (95%CI = 0.36–0.67)			

RAPID3 = Routine Assessment of Patient Index Data 3; CDAI = Clinical Disease Activity Index; SDAI = Simple Disease Activity Index; DAS28 (ESR) = Disease Activity Score 28; Bold: number of coincidental categories among analyzed scores; Italic: total number in respective categories

In the individual analysis, a moderate quadratic weighted kappa of 0.4 (95%CI 0.3–0.5) was obtained. When the grouped analysis was performed for RAPID3 with DAS28 (ESR) a moderate kappa of 0.5 (95%CI 0.3–0.6) and slight agreement between CDAI and SDAI was observed (Table 4).

In the analysis of RAPID3 to predict moderate and high activity by CDAI, SDAI and DAS28, a sensitivity of 98%, specificity of 49% and a high negative predictive value (92%), was found for the gold standard DAS28, this was similar for CDAI and SDAI. (Table 5) The finding of negative post-test probability of 8% is also reinforced, which means that among people identified as in remission/low disease activity, only 8 out of 100 patients would actually have moderate/high disease activity.

**Table 5:** Sensitivity, Specificity and Negative Post-Test Probability of RAPID3 to predict moderate and high activity by CDAI, SDAI and DAS28

Indexes	Sensitivity	Specificity	NPP
	% (95%CI)	% (95%CI)	% (95%CI)
CDAI	97 (89–99)	38 (26–52)	8 (1–28)
SDAI	97 (89–99)	40 (27–54)	8 (1–28)
DAS28 (ESR)	97 (90–99)	48 (33–64)	8 (1–28)

95%CI = 95% Confidence interval; NPP = negative post-test probability; RAPID3 = Routine Assessment of Patient Index Data 3; CDAI = Clinical Disease Activity Index; SDAI = Simple Disease Activity Index; DAS28 (ESR) = Disease Activity Score 28

**Fig. 1:**
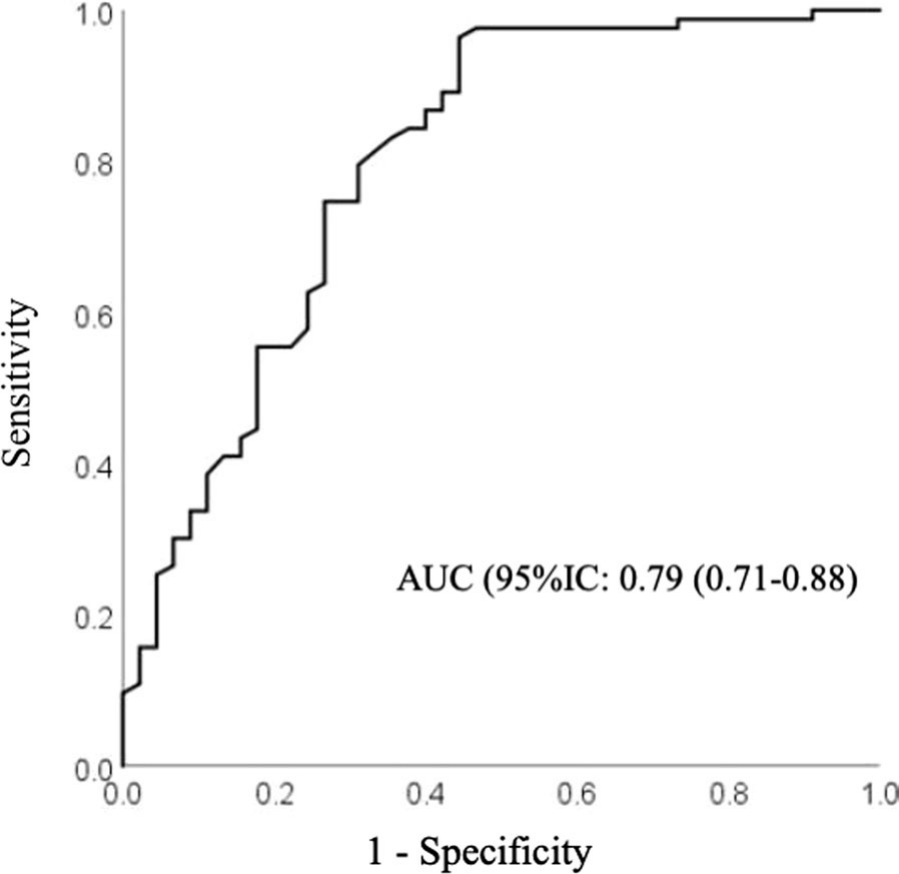
ROC curve for the performance of RAPID3 in relation to the gold standard DAS28

The ROC curve analysis of RAPID3 in relation to the gold standard DAS28 and its area under the curve (AUC) measurement was performed and is illustrated in Fig. 1, with RAPID3 showing an AUC of 0.79 (95%CI 0.71–0.88) and a cutoff point of 0.5 for test positivity or negativity, showing RAPID3 as a tool with good diagnostic accuracy.

In this study, the Boolean remission criteria was met in only 2 patients, not being relevant for analysis. According to the definition, a patient is considered to be in remission by these criteria when the painful joint count, the swollen joint count, the patient's global assessment of disease activity, and the C-reactive protein (CRP) each do not exceed score of one [26].

In our study population, 57% of patients had a RADAI ≥ 16 and 56% a FAST3 ≥ 2, most likely reflecting the higher composite disease activity scores.

Regarding the ease of use, 90% of patients considered the electronic MDHAQ easy to fill and understand, and 93% considered it a good tool to assess their symptoms.

## Discussion

This study was the first formal validation of the RAPID3 in a Brazilian population of patients with RA. Adaptations and reports identified in the conceptual equivalence process were then submitted for consideration by the copyright holder.

The individual evaluation of the RAPID3 score items by CVI was perceived as predominantly homogeneous and of excellent performance among health professionals, especially among rheumatologists. RAPID3 showed test–retest reliability and adequate internal consistency.

The correlation of scores obtained with MDHAQ/RAPID3 demonstrate an adequate association with the DAS28 gold standard. There was a correlation between the different disease activity scores, with good agreement between the composite disease activity indices in predicting moderate/high disease activity. These findings were similar to previous studies carried out both in clinical research participants and in different populations of rheumatologic patients [8, 19, 21, 27–31].

Patient’s global assessments of general status and pain intensity obtained a proportional correlation with the higher the disease activity score, for all analyzed composite disease indices. FAST3 includes the assessment of patient self-reported painful joints (RADAI) in its calculation, both of which are important in the assessment of unaltered RAPID3 scores, which may occur as a result of overlapping RA comorbidities such as fibromyalgia and osteoarthritis. Clinical improvement is less likely in patients with RA and fibromyalgia overlap, a situation that can lead to unnecessary escalation of disease course-modifying treatment if not properly evaluated. Thus, FAST3 can be used to screen for fibromyalgia, a comorbidity that affects around 20–40% of patients with RA [19, 32].

It is observed that the clinical validation sample of our study showed a significant portion of patients in moderate and high activity, and few patients were in remission. This was due to the coincident period of data collection and prioritization of appointments during the resumption of outpatient care at HCPA after the restrictions imposed by the COVID-19 pandemic.

It is possible that this fact has an impact on the kappa estimative, especially the unweighted kappa of the study, as the sample is not well distributed among patients with disease in remission, low, moderate and high activity.

Likewise, due to this profile of the study population, it was not possible to judge the ACR/EULAR Boolean remission criterion between the indices, as only two patients met the criterion. These criteria are strict and difficult to achieve in patients with long-term RA and with established damage, as in the case of our sample (median 13 years of disease duration).

Despite the limitation in the study in relation to a small sample of patients in remission/low activity, this fact did not significantly impact the evaluation of the RAPID3 instrument as a screening method, and it presented similar intraclass correlation indices found in previously conducted studies. In the validation study of RAPID3 for the Japanese population, Yokogawa et al. obtained a correlation index of 0.76 for the CDAI and 0.55 for the DAS28 [21]. In another study by Kim and colleagues, the kappa value for CDAI, SDAI, and DAS28 disease activity scores ranged around 0.40 [33].

Another limitation was the inability to assess changes in disease activity scores, due to the study design itself. Complementary studies with temporal follow-up of patients will be able to predict the correlation, as well as the sensitivity and specificity for changes in clinical scores and risk of flare identifiable by RAPID3 and other tools in the context of telehealth [9, 34].

In consideration for the use of RAPID3 as a clinical screening tool, it is supported by the fact that it had a high sensitivity, adequate specificity and a high negative predictive value, attributes expected for a test tool for population screening [35, 36]. This usefulness of RAPID3 is of paramount importance in the current context, as it allows the identification of the population of rheumatologic patients at risk, favoring the screening and prioritization of these patients in waiting demand or in need of early referral of these patients to tertiary care.

## Conclusions

The use of MDHAQ/RAPID3 associated with traditional clinical measures can allow the maintenance of remote monitoring with a clinical activity score performance comparable to the DAS28 gold standard, in addition to allowing an understanding of the impact on the health dynamics of rheumatologic patients in the context of telehealth, both in terms of care and clinical research [9, 27].

A self-reported assessment tool by the patient, such as RAPID3, also allows for a shared dynamic between physician and patient regarding the decisions of their clinical follow-up, improving adherence to treatment and being an important source of monitoring of adverse effects and effectiveness of the therapeutic measures instituted [37–39].

## Data Availability

Supporting data might be available as needed.

## Abbreviations

ACR: American College of Rheumatology
AUC: Area under the curve
CDAI: Clinical Disease Activity Index
CRP: C-reactive protein
CVI: Content Validation Index
DAS28: Disease Activity Score 28-joints
ESR: Erythrocyte sedimentation rate
EULAR: European Alliance of Associations for Rheumatology
FAST3: Fibromyalgia Assessment Screening Test
HAQ: Health Assessment Questionnaire
MDHAQ: Multidimensional Health Assessment Questionnaire
PSYCH3: Psychological Index 3
RADAI: Rheumatoid Arthritis Disease Activity Index
RAPID3: Routine Assessment of Patient Index Data 3
SDAI: Simplified Disease Activity Index
SPSS: Statistical package for social sciences
SUS: System Usability Scale
VAS: Visual analogue scale
